# Mental Health Status of Cisgender and Gender-Diverse Secondary School Students in China

**DOI:** 10.1001/jamanetworkopen.2020.22796

**Published:** 2020-10-27

**Authors:** Yuanyuan Wang, Hui Yu, Yong Yang, Jack Drescher, Ronghua Li, Weijia Yin, Renjie Yu, Shuilan Wang, Wei Deng, Qiufang Jia, Kenneth J. Zucker, Runsen Chen

**Affiliations:** 1 National Clinical Research Center for Mental Disorders, Department of Psychiatry, and China National Technology Institute on Mental Disorders, The Second Xiangya Hospital of Central South University, Changsha, China; 2 Division of Psychology, Faculty of Health and Life Sciences, De Montfort University, Leicester, United Kingdom; 3 Suzhou Guangji Hospital, Affiliated Guangji Hospital of Soochow University, Soochow University, Suzhou, China; 4 Postdoctoral Program in Psychotherapy and Psychoanalysis, New York University, New York; 5 Department of Psychiatry, Columbia University, New York, New York; 6 Mental Health Center, West China Hospital of Sichuan University, Chengdu, China; 7 Department of Psychiatry, University of Toronto, Toronto, Ontario, Canada; 8 Department of Psychiatry, The Affiliated Brain Hospital of Nanjing Medical University, Nanjing, China

## Abstract

**Importance:**

Transgender or gender nonconforming (TGNC) adolescents face a wide range of physical and mental health concerns. However, there has been no school-based study to explore the prevalence and mental health status of these adolescents in mainland China.

**Objectives:**

To assess the mental well-being of TGNC adolescents in China by comparing them with their cisgender peers.

**Design, Setting, and Participants:**

This was a cross-sectional survey study. Of the 12 354 adolescents who completed the questionnaire, 246 did not specify to which sex they identified and were therefore excluded from further analysis. Therefore, a total of 12 108 adolescents were included from 18 secondary schools in Suzhou city, China, from June 2019 to July 2019. Participants provided consent and answered questions on sex assigned at birth and gender identity.

**Main Outcomes and Measures:**

All participants completed questionnaires, including the Patient Health Questionnaire for the measurement of depressive symptoms, a generalized anxiety disorder screening, the Pittsburgh Sleep Quality Index, and a self-harm and suicide risk checklist. The frequency of being bullied at school was also measured. Participants who reported their perceived gender as the opposite of their assigned sex at birth were classified as *transgender*, those who identified as neither male nor female were classified as *nonbinary*, and those who were not sure about their perceived gender were classified as *questioning*. All of these participants were categorized as TGNC adolescents.

**Results:**

A total of 12 108 adolescents (mean [SD] age, 15.8 [1.0] years; 6518 [53.8%] assigned male at birth [AMAB]) participated in the study. Of the 6518 participants AMAB in the sample, 5855 (89.8%) were classified as cisgender boys, 208 (3.2%) as transgender girls (transgender youth who perceive their current gender identity to be female), 138 (2.1%) as nonbinary youth AMAB, and 317 (4.9%) as questioning youth AMAB. Of the 5590 participants assigned female at birth (AFAB), 4142 (74.1%) were classified as cisgender girls, 861 (15.4%) as transgender boys (transgender youth who perceive their current gender identity to be male), 112 (2.0%) as nonbinary youth AFAB, and 475 (8.5%) as questioning youth AFAB. Compared with cisgender adolescents, TGNC adolescents reported significantly higher health concerns including lower overall health (*t*_11 872 _= –7.36; *P* < .001), poorer sleep (*t*_11 683 _= 10.49; *P* < .001), higher depression and anxiety symptoms (*t*_11 830 _= 12.43 and *t*_11 847 _= 11.47, respectively; *P* < .001), and higher rate of self-harm and suicide ideation (*t*_11 860 _= 12.22; *P* < .001). The TGNC youth who were AMAB were also more likely to be bullied at school than cisgender boys (transgender girls: odds ratio [OR], 2.34 [95% CI, 1.64-3.33]; nonbinary youth AMAB: OR, 1.97 [95% CI, 1.23-3.16]; and questioning youth AMAB: OR, 1.95 [95% CI, 1.43-2.67]). The TGNC groups also reported significantly greater amounts of thoughts of self-harm (transgender girls: OR, 3.06 [95% CI, 2.24-4.19]; transgender boys: OR, 4.06 [95% CI, 3.47-4.74]; nonbinary youth AMAB: OR, 2.86 [95% CI, 1.93-4.23]; nonbinary youth AFAB: OR, 3.71 [95% CI, 2.46-5.59]; questioning youth AMAB: OR, 2.61 [95% CI, 1.98-3.44]; and questioning youth AFAB: OR, 3.35 [95% CI, 2.70-4.16]), thoughts of suicide (transgender girls: OR, 3.93 [95% CI, 2.88-5.38]; transgender boys: OR, 3.71 [95% CI, 3.10-4.21]; nonbinary youth AMAB: OR, 3.13 [95% CI, 2.11-4.63]; nonbinary youth AFAB: OR, 3.78 [95% CI, 2.50-5.71]; questioning youth AMAB: OR, 2.53 [95% CI, 1.93-3.33]; and questioning youth AFAB: OR, 3.94 [95% CI, 3.17-4.88]), suicide plan formation (transgender girls: OR, 4.44 [95% CI, 2.88-6.83]; transgender boys: OR, 2.66 [95% CI, 2.03-3.50]; nonbinary youth AMAB: OR, 5.36 [95% CI, 3.22-8.93]; nonbinary youth AFAB: OR, 4.06 [95% CI, 2.25-7.30]; and questioning youth AFAB: OR, 2.36 [95% CI, 1.63-3.43]), deliberate self-harm during the last month (cisgender girls: OR, 1.49 [95% CI, 1.33-1.68]; transgender girls: OR, 2.74 [95% CI, 1.93-3.91]; transgender boys: OR, 3.06 [95% CI, 2.57-3.66]; nonbinary youth AMAB: OR, 2.56 [95% CI, 1.66-3.94]; nonbinary youth AFAB: OR, 3.06 [95% CI, 1.95-4.81]; questioning youth AMAB: OR, 2.14 [95% CI, 1.56-2.92]; and questioning youth AFAB: OR, 2.53 [95% CI, 2.00-3.01]), and attempts of suicide (transgender girls: OR, 4.35 [95% CI, 2.88-6.56]; transgender boys: OR, 2.92 [95% CI, 2.26-3.76]; nonbinary youth AMAB: OR, 3.94 [95% CI, 2.36-6.55]; nonbinary youth AFAB: OR, 3.06 [95% CI, 1.67-5.63]; questioning youth AMAB: OR, 2.61 [95% CI, 1.73-3.94]; and questioning youth AFAB: OR, 1.93 [95% CI, 1.33-2.81]) compared with cisgender boys.

**Conclusions and Relevance:**

Results of this cross-sectional survey study suggest poor mental health status among TGNC adolescents in China; in addition, findings suggest a compelling need for researchers, practitioners, and policy makers to address these mental health problems. Particular school-based interventions are recommended to support the mental health well-being of TGNC adolescents.

## Key Points

**Question:**

What factors are associated with mental health outcomes among transgender or gender nonconforming (TGNC) adolescents in China?

**Findings:**

In this cross-sectional survey study of 12 108 adolescents in China, TGNC adolescents reported poorer mental health, including a higher level of anxiety, depression, and sleeping problems, compared with their cisgender counterparts. TGNC adolescents were more likely to report being bullied, experienced more suicidal thoughts and behaviors, and harmed themselves more often than cisgender adolescents.

**Meaning:**

Compared with cisgender adolescents, TGNC adolescents in China appear to face a higher level of various mental health challenges.

## Teaser

This cross-sectional study uses data from questionnaires completed by adolescents in secondary schools in Suzhou city, China, to investigate the mental health challenges of youth identifying as transgender and gender nonconforming compared with cisgender adolescents.

## Introduction

*Transgender* is an umbrella term that refers to individuals whose gender identity is incongruent with their sex assigned at birth.^1^ Gender roles vary across societies, although the majority of people conform to their culture’s gender norms.^2^ Those who do not tend to be either rendered invisible or vulnerable to harassment and bullying.^3^ Today, the number of transgender adolescents appears to be increasing around the world, and there is an increasing number of adolescents being referred to specialized gender identity clinics.^4,5^ The decreased stigmatization of sexual and gender minorities, the availability of information on the internet, and increased media attention may be increasing the likelihood of adolescents self-identifying as transgender or gender nonconforming (TGNC).

Transgender and gender nonconforming individuals experience various mental health challenges. Gender dysphoria in the *Diagnostic and Statistical Manual of Mental Disorders* (Fifth Edition) is the psychiatric diagnosis for individuals who have a strong desire to be of the other gender and experience the incongruence as distressing.^6,7,8^ Research has shown that a significant proportion of adolescents with gender dysphoria have a history of other psychiatric diagnoses.^5,9^ There are also a number of studies reporting a high percentage of suicidal ideation, self-harm, and suicide attempts in adolescents with gender dysphoria.^10,11,12,13^ Compared with their cisgender peers, TGNC youth are more likely to report mental health problems such as anxiety, depression, and suicidal ideation.^1,14^ Moreover, within the school environment, transgender adolescents are vulnerable to harassment and bullying.^15^

Transgender and gender nonconforming youth are a diverse group and face considerable obstacles to health and well-being.^13,16,17,18,19^ To our knowledge, there have been no school-based surveys to examine the prevalence and mental health status of TGNC adolescents in mainland China. In surveying a nonclinical sample of adolescents, this study aimed to compare the mental health status of TGNC and cisgender adolescents. It also investigated mental health disparities among transgender youth subgroups.

## Methods

### Study Design and Setting

This was a cross-sectional survey study. Students attending 18 public secondary schools (grades 7-11) in Suzhou, a metropolitan city in China, were invited to participate. None of the schools declined to participate in this study. Data were collected between June 2019 and July 2019. We used cluster sampling of 18 public middle and high schools in one of the districts in Suzhou city. Ethical approval was obtained from Suzhou Guangji Hospital. School teachers led the recruitment, and it was made clear to potential participants that participation was voluntary and that there were no adverse consequences if they refused to participate or later withdrew. Students, who provided written consent, were informed that their school teachers were not able to access the completed questionnaires. This study followed the Strengthening the Reporting of Observational Studies in Epidemiology (STROBE) reporting guideline.

### Participants and Exposure Variable

All students in the 18 schools received information about the study. A total of 12 354 questionnaires were returned, and the response rate was 83.2%. The student’s gender identity was measured by 2 questions: (1) biological sex: “What was your biological sex assigned at birth (choose from male or female)?” and (2) perceived gender: “What do you perceive your gender to be (choose from male, female, neither, or not sure)?” Students were categorized into 8 gender groups based on sex assigned at birth and perceived gender. Those who identified their present gender as congruent with their sex assigned at birth were classified as *cisgender*. Those who identified their gender clearly as the opposite of their sex assigned at birth were classified as *transgender*; those who identified as neither male nor female were classified as *nonbinary*; and those who were not sure about their gender were classified as *questioning*. These 4 gender groups were further subdivided as a function of sex assigned at birth: assigned male at birth (AMAB) or assigned female at birth (AFAB). This method of categorization using 2 questions has been used to assess gender identity in previous Chinese and European studies.^20,21^

### Dependent Variables

#### Overall Physical Health and Patient Health Questionnaire 

Participants were asked about their physical health using item 1 from the 36-Item Short Form Survey Instrument (“In general, would you say your health is…?”),^22^ and participants could choose an answer that ranged from poor to excellent. The Patient Health Questionnaire 9 (PHQ-9)^23^ was used to measure the severity of depressive symptoms. There were 9 items (eg, “little interest or pleasure in doing things”). Participants were asked to rate how often they had been bothered by any of the problems over the previous 2 weeks. The total score of the 9 items was calculated.

#### Generalized Anxiety Disorder Screening

The Generalized Anxiety Disorder 7-item scale (GAD-7)^24^ was used to measure anxiety symptoms. There were 7 items (eg, “worrying too much about different things”). Similar to the PHQ-9, participants were asked to report any of the problems over the previous 2 weeks. The total score of the 7 items was calculated.

#### Chinese Version of the Pittsburgh Sleep Quality Index 

The 10-item Chinese version of the Pittsburgh Sleep Quality Index (CPSQI)^25^ was used to evaluate participants’ sleep quality in the past month (eg, “it was difficult to go to sleep”). Participants were asked to rate each item on the frequency of experiencing these sleep problems. The total score of the 10 items was calculated.

#### Frequency of Being Bullied at School

The frequency of being bullied at school was questioned with 1 item: “How often have you been bullied at school in this academic year?”^26^ In the analyses, responses were dichotomized as being bullied or not being bullied at school.

#### Suicide Risk Checklist

Self-harm and suicidality were assessed by asking participants to indicate their behaviors and thoughts. Items used by Chen et al^27^ were modified, and there were 5 binary yes-or-no questions that asked about participants’ self-harm thoughts and behavior, suicidal thoughts, suicide plan, and suicide attempts. Self-harm thoughts and behavior were measured using the questions, “Did you want to harm yourself in the last month?” and “Did you deliberately harm yourself in the last month?” A suicidal thought was measured by, “Did you think about suicide in the last month?” Suicide plan was measured by, “Did you have a suicide plan in the last month?” Participants were asked whether, in their lifetime, they had ever made a suicide attempt.

As in Chen et al,^27^ 5 specific questions were used to calculate a self-harm and suicidal ideation score: (1) “Did you think you would be better off dead or wish you were dead in the last month?” If yes, the score was 1; (2) if yes to self-harm thoughts in the last month, the score was 2; (3) if yes to suicide thoughts in the last month, the score was 4; (4) if yes to a suicide plan in the last month, the score was 10; and (5) if participants ever attempted suicide in the past, the score was 4. Then, an overall score of self-harm and suicidal inclination was calculated for each participant.

### Statistical Analysis

All analyses were carried out from December 2019 to August 2020 using Mac R software, version 4.0.1 (R Foundation). Given the large sample size, *P* < .01 was taken to indicate statistical significance in all analyses (some analyses adopted a more stringent *P* < .001 criterion). All *P* values were 2-sided. Missing data were deleted listwise in all analyses.

To assess the difference in the physical and mental health status among the different sex and TGNC groups, a series of linear mixed-model analyses were tested with participants’ self-reported overall health, depression, anxiety, sleep quality, frequency of being bullied, and suicidal ideation as the outcome variables, whereas sex (male as the baseline), gender identity (cisgender youth as the baseline), and their interaction were the explanatory variables. School was added in as a random-effect term to account for the potential difference of school policy and culture affecting the tolerance for gender minority groups.

To assess the possible associated risk of being bullied and tendency toward self-harm and suicidal ideation in association with the different gender identity groups, a series of mixed-effects logistic regressions were conducted with the 8-gender identity group category used as the exposure variable (the cisgender boys were treated as the reference group) and school as a random-effects term. The dichotomous (yes or no) response to questions regarding self-harm thoughts and self-harm actions, suicidal thoughts, suicide plans, suicide attempts, and being bullied were used as outcome variables. The same regressions were also conducted with cisgender girls as the reference group, and the results are available in eTable 1 in the Supplement.

## Results

### Participants

All students who completed the questionnaire (6688 boys and 5666 girls) answered the question about their sex assigned at birth; however, there were 170 boys (2.5%) and 76 girls (1.3%) who did not specify to which sex they identified and were therefore excluded from further analysis. A total of 12 108 adolescents (mean [SD] age, 15.8 [1.0] years; 6518 [53.8%] AMAB) participated in the study. Of the 6518 respondents AMAB in the sample, 5855 (89.8%) were classified as cisgender boys, 208 (3.2%) as transgender girls (transgender youth who perceive their current gender identity to be female), 138 (2.1%) as nonbinary youth AMAB, and 317 (4.9%) as questioning youth AMAB. Of the 5590 participants AFAB, 4142 (74.1%) were classified as cisgender girls, 861 (15.4%) as transgender boys (transgender youth who perceive their current gender identity to be male), 112 (2.0%) as nonbinary youth AFAB, and 475 (8.5%) as questioning youth AFAB.

### Descriptive Statistics

The Cronbach α for each measure was as follows: 0.93 for the PHQ-9, 0.94 for GAD-7, and 0.83 for the CPSQI. The mean (SD) values of the health-related indicators by gender groups are shown in Table 1.

**Table 1.** Mean (SD) Values of Health-Related Variables by Gender Groups

Variable	No.	Mean (SD)^a^					
		Age	PHQ-9 (0-27)	GAD-7 (0-21)	CPSQI (10-40)	Overall health (1-5)	Self-harm and suicidal ideation (0-21)
Cisgender							
Boys	5855	14.92 (1.46)	4.63 (5.63)	4.43 (4.72)	13.78 (4.83)	4.00 (0.82)	1.25 (3.60)
Girls	4142	14.95 (1.48)	5.13 (5.83)	5.33 (4.87)	14.07 (4.43)	3.87 (0.76)	1.54 (3.79)
Transgender							
Girls (AMAB)	208	15.26 (1.62)	8.03 (7.78)	7.00 (6.30)	16.50 (7.48)	3.84 (0.93)	4.03 (6.44)
Boys (AFAB)	861	14.96 (1.47)	8.28 (7.35)	7.26 (5.81)	15.94 (5.59)	3.72 (0.82)	3.51 (5.47)
Nonbinary							
AMAB	138	15.20 (1.53)	7.78 (7.95)	7.01 (6.78)	15.71 (6.00)	3.71 (0.98)	3.93 (6.69)
AFAB	112	15.20 (1.47)	8.45 (7.46)	7.51 (6.01)	15.79 (6.25)	3.65 (0.78)	3.86 (5.82)
Questioning							
AMAB	317	14.89 (1.51)	7.81 (7.27)	6.85 (6.05)	15.80 (5.40)	3.66 (0.86)	2.57 (4.72)
AFAB	475	14.91 (1.47)	7.65 (6.49)	6.74 (5.22)	16.08 (5.47)	3.59 (0.73)	3.17 (5.12)
Total	12 108	14.95 (1.68)	5.36 (6.12)	5.18 (5.07)	14.26 (4.95)	3.90 (0.81)	1.72 (4.13)

Abbreviations: AFAB, assigned female at birth; AMAB, assigned male at birth; CPSQI, Chinese version of the Pittsburgh Sleep Quality Index; GAD-7, Generalized Anxiety Disorder 7-item scale; PHQ-9, Patient Health Questionnaire 9.

^a^ The numerical range of the measured scales is listed with each measure.

### Physical and Mental Health Status and Frequency of Being Bullied Among Sex and Gender Identity Groups

The linear mixed-model analysis results are shown in Table 2. Youth who were AFAB reported a significantly lower level of overall health (t_11 866_ = −7.91, *P* < .001), higher depression (t_11 827_ = 3.88, *P* < .001) and anxiety symptoms (t_11 845_ = 8.71, p < .001), higher sleep problems (t_11 676_ = 2.79, *P* = .005), and higher suicide ideation (t_11 848_ = 3.44, *P* < .001), but a lower frequency of being bullied at school than youth who were AMAB (t_12 042_ = −7.79, *P* < .001). On the other hand, in comparison with the cisgender youth, TGNC youth reported a significantly lower overall health (*t*_11 872 _= −7.36; *P* < .001), higher depression (*t*_11 830 _= 12.43; *P* < .001) and anxiety symptoms (*t*_11 847 _= 11.47; *P* < .001), higher sleep problems (*t*_11 683 _= 10.49; *P* < .001), higher frequency of being bullied at school (school (t_12 050_ = 10.07, *P* < .001), and higher suicide ideation (*t*_11 860 _= 12.22; *P* < .001). The sex and gender identity interaction was not significant for all outcome variables except for the frequency of being bullied (t_12 044_ = −4.80, *P* < .001) (Figure).

**Table 2.** Linear Mixed-Model Analysis of the Association of Sex and Gender Identity With Health-Related Variables, Frequency of Being Bullied at School, and Suicide Ideation

Exposure variable	Unstandardized coefficient (95% CI)	*t* Value^a^	*P* value^b^
Outcome variable = overall health			
Sex^c^	−0.13 (−0.17 to −0.09)	−7.91	<.001
Gender identity^d^	−0.25 (−0.31 to −0.19)	−7.63	<.001
Sex × gender identity	0.07 (−0.01 to 0.15)	1.64	.10
Outcome variable = depression			
Sex^c^	0.47 (0.23 to 0.70)	3.88	<.001
Gender identity^d^	3.08 (2.59 to 3.57)	12.43	<.001
Sex × gender identity	−0.21 (−0.82 to 0.40)	−0.69	.49
Outcome variable = anxiety			
Sex^c^	0.88 (0.68 to 1.08)	8.71	<.001
Gender identity^d^	2.35 (1.96 to 2.74)	11.47	<.001
Sex × gender identity	−0.63 (−1.12 to −0.14)	−2.49	.01
Outcome variable = sleep problems			
Sex^c^	0.28 (0.08 to 0.48)	2.79	.005
Gender identity^d^	2.14 (1.75 to 2.53)	10.49	<.001
Sex × gender identity	−0.23 (−0.72 to 0.26)	−0.91	.36
Outcome variable = frequency of being bullied			
Sex^c^	−0.06 (−0.08 to −0.04)	−7.79	<.001
Gender identity^d^	0.17 (0.13 to 0.21)	10.07	<.001
Sex × gender identity	−0.10 (−0.14 to −0.06)	−4.80	<.001
Outcome variable = suicide ideation			
Sex^c^	0.28 (0.12 to 0.44)	3.44	<.001
Gender identity^d^	2.05 (1.72 to 2.38)	12.22	<.001
Sex × gender identity	−0.17 (−0.58 to 0.24)	−0.81	.42

Abbreviation: TGNC, transgender or gender nonconforming.

^a^ School was added as a random-effect term.

^b^ *P* < .01 is the cutoff criterion.

^c^ Sex, 0 = boys; 1 = girls.

^d^ Gender identity, 0 = cisgender youth; 1 = TGNC youth.

**Figure.**
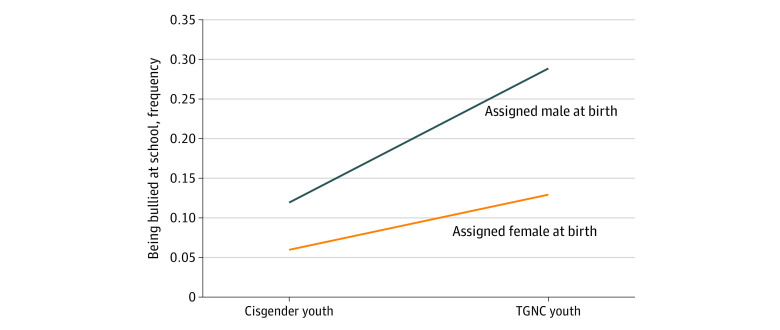
Sex and Gender Identity Interaction With Bullying Frequency at School The frequency of being bullied at school indicated in each group is the estimated mean in the linear model. TGNC indicates transgender or gender nonconforming.

### Association of Gender Groups With Risk of Self-Harm, Suicide, and Being Bullied 

As shown in Table 3, compared with cisgender boys, the gender minority groups as well as cisgender girls had a significantly higher likelihood of reporting self-harm thoughts (cisgender girls: odds ratio [OR], 1.48; 95% CI, 1.31-1.66) and suicide thoughts (cisgender girls: OR, 1.42; 95% CI, 1.26-1.60) as well as actual deliberate self-harm (cisgender girls: OR, 1.49; 95% CI, 1.33-1.68) during the last month. Compared with cisgender boys, all gender minority groups reported a significantly higher incidence of past suicide attempts (transgender girls: OR, 4.35; 95% CI, 2.88-6.56; transgender boys: OR, 2.92; 95% CI, 2.26-3.76; nonbinary youth AMAB: OR, 3.94; 95% CI, 2.36-6.55; nonbinary youth AFAB: OR, 3.06; 95% CI, 1.67-5.63; questioning youth AMAB: OR, 2.61; 95% CI, 1.73-3.94; and questioning youth AFAB: OR, 1.93; 95% CI, 1.33-2.81); all gender minority groups except questioning youth AMAB were significantly more likely to report having a suicide plan in the past month (transgender girls: OR, 4.44; 95% CI, 2.88-6.83; transgender boys: OR, 2.66; 95% CI, 2.03-3.50; nonbinary youth AMAB: OR, 5.36; 95% CI, 3.22-8.93; nonbinary youth AFAB: OR, 4.06; 95% CI, 2.25-7.30; and questioning youth AFAB: OR, 2.36; 95% CI, 1.63-3.43). In addition, cisgender girls were less likely to be bullied at school in comparison with cisgender boys (cisgender girls: OR, 0.49; 95% CI, 0.41-0.58), but transgender girls (AMAB), nonbinary youth AMAB, and questioning youth AMAB were significantly more likely to be bullied than cisgender boys (transgender girls: OR, 2.34; 95% CI, 1.64-3.33; nonbinary youth AMAB: OR, 1.97; 95% CI, 1.23-3.16; and questioning youth AMAB: OR, 1.95; 95% CI, 1.43-2.67).

**Table 3.** Mixed-Effects Logistic Regression Results With Cisgender Boys as the Reference Group: Association Between Gender Groups and Self-Harm, Suicidal Thoughts and Actions, and Being Bullied at School

Question	Answered no, No. (%)	Answered yes, No. (%)	Odds ratio (95% CI)^a^
**Did you want to harm yourself in the last month?**			
Cisgender			
Boys	5151 (89.0)	634 (11.0)	1 [Reference]
Girls	3478 (84.6)	634 (15.4)	1.48 (1.31-1.66)
Transgender			
Girls (AMAB)	149 (72.7)	56 (27.3)	3.06 (2.24-4.19)
Boys (AFAB)	564 (66.5)	284 (33.5)	4.06 (3.47-4.74)
Nonbinary youth			
AMAB	100 (74.1)	35 (25.9)	2.86 (1.93-4.23)
AFAB	77 (68.7)	35 (31.3)	3.71 (2.46-5.59)
Questioning			
AMAB	236 (75.4)	77 (24.6)	2.61 (1.98-3.44)
AFAB	334 (71.2)	135 (28.8)	3.35 (2.70-4.16)
**Did you deliberately harm yourself in the last month?**			
Cisgender			
Boys	5243 (91.1)	515 (8.9)	1 [Reference]
Girls	3578 (87.2)	526 (12.8)	1.49 (1.33-1.68)
Transgender			
Girls (AMAB)	162 (79.0)	43 (21.0)	2.74 (1.93-3.91)
Boys (AFAB)	652 (76.9)	196 (23.1)	3.06 (2.57-3.66)
Nonbinary youth			
AMAB	108 (80.0)	27 (20.0)	2.56 (1.66-3.94)
AFAB	86 (76.8)	26 (23.2)	3.06 (1.95-4.81)
Questioning			
AMAB	259 (82.7)	54 (17.3)	2.14 (1.56-2.92)
AFAB	376 (80.2)	93 (19.8)	2.53 (2.00-3.01)
**Did you think about suicide in the last month?**			
Cisgender			
Boys	5158 (89.2)	623 (10.8)	1 [Reference]
Girls	3501 (85.4)	598 (14.6)	1.42 (1.26-1.60)
Transgender			
Girls (AMAB)	139 (67.8)	66 (32.2)	3.93 (2.88-5.38)
Boys (AFAB)	581 (68.9)	262 (31.1)	3.71 (3.10-4.21)
Nonbinary youth			
AMAB	97 (72.4)	37 (27.6)	3.13 (2.11-4.63)
AFAB	76 (68.5)	35 (31.5)	3.78 (2.50-5.71)
Questioning			
AMAB	238 (76.3)	74 (23.7)	2.53 (1.93-3.33)
AFAB	317 (68.2)	148 (31.8)	3.94 (3.17-4.88)
**Did you have a suicide plan and prepare to die in the last month?**			
Cisgender			
Boys	5572 (96.8)	187 (3.2)	1 [Reference]
Girls	3974 (96.8)	131 (3.2)	0.98 (0.77-1.24)
Transgender			
Girls (AMAB)	179 (87.3)	26 (12.7)	4.44 (2.88-6.83)
Boys (AFAB)	779 (91.8)	70 (8.2)	2.66 (2.03-3.50)
Nonbinary youth			
AMAB	115 (85.2)	20 (14.8)	5.36 (3.22-8.93)
AFAB	99 (88.4)	13 (11.6)	4.06 (2.25-7.30)
Questioning			
AMAB	296 (94.6)	17 (5.4)	1.73 (1.04-2.88)
AFAB	434 (92.7)	34 (7.3)	2.36 (1.63-3.43)
**Have you ever attempted suicide in the past?**			
Cisgender			
Boys	5538 (96.2)	217 (3.8)	1 [Reference]
Girls	3928 (96.7)	177 (4.3)	1.15 (0.94-1.40)
Transgender			
Girls (AMAB)	175 (85.4)	30 (14.6)	4.35 (2.88-6.56)
Boys (AFAB)	761 (89.7)	87 (10.3)	2.92 (2.26-3.76)
Nonbinary youth			
AMAB	116 (86.6)	18 (13.4)	3.94 (2.36-6.55)
AFAB	100 (89.3)	12 (10.7)	3.06 (1.67-5.63)
Questioning			
AMAB	284 (90.7)	29 (9.3)	2.61 (1.73-3.94)
AFAB	436 (93.0)	33 (7.0)	1.93 (1.33-2.81)
**Have you ever been bullied at school in this academic year?**			
Cisgender			
Boys	5316 (91.1)	522 (8.9)	1 [Reference]
Girls	3949 (96.5)	187 (4.5)	0.49 (0.41-0.58)
Transgender			
Girls (AMAB)	169 (81.2)	39 (18.8)	2.34 (1.64-3.33)
Boys (AFAB)	779 (90.6)	81 (9.4)	1.05 (0.77-1.44)
Nonbinary youth			
AMAB	115 (83.9)	22 (16.1)	1.97 (1.23-3.16)
AFAB	104 (92.9)	8 (7.1)	0.78 (0.38-1.62)
Questioning			
AMAB	265 (83.9)	51 (16.4)	1.95 (1.43-2.67)
AFAB	432 (90.9)	43 (9.1)	1.02 (0.73-1.42)

Abbreviations: AFAB, assigned female at birth; AMAB, assigned male at birth.

^a^ School was added as a random-effects term.

## Discussion

To our knowledge, this is the first study to assess the occurrence of mental health problems in TGNC adolescents in mainland China. The findings suggest that TGNC students reported more difficulties and problems on all of the measures. Results suggest that TGNC adolescents reported significantly higher health-related problems than cisgender adolescents, including higher depressive and anxiety symptoms as well as poorer overall health and sleep quality. In addition, compared with cisgender adolescents, TGNC adolescents reported a significantly higher frequency of being bullied at school, having self-harm thoughts, deliberately participating in self-harm behavior, having suicidal thoughts, making suicide plans, and attempting suicide. We also found a relatively high prevalence rate of those who identify as TGNC in the current sample.

Consistent with previous research, self-harm thoughts and behaviors were endorsed more frequently in the TGNC group.^28^ Our study also explored factors associated with risk behaviors, including self-harm and suicidal ideation. Not surprisingly, depressive symptoms, lower overall physical health, and being bullied at school were all positively associated with risk of self-harm behavior and suicide attempts. Similarly, a previous study also reported that bullying in gender dysphoric adolescents was significantly associated with self-reported behavioral and emotional problems.^29^ Such distress could be both from the gender dysphoria per se and from external factors, such as discrimination and victimization in the social environment.^29,30^

A previous survey in New Zealand with a sample of 8166 adolescents found that 1.2% of participants self-identified as transgender, and 2.5% of participants self-reported as gender nonconforming.^16^ Also, a study conducted in Taiwan^31^ showed that gender dysphoria was more prevalent in female young adults (7.3%) than in male young adults (1.9%). The current study found a much higher prevalence of a TGNC identity. The method used to classify the adolescents to different gender identity categories was established in a few previous studies.^21^ In particular, participants were asked which gender they perceived themselves to be; when the perception was incongruent with their sex assigned at birth, they were classified as TGNC. This method is different from those that ask the participants to choose their gender identity directly from a choice of male, female, transgender, or not sure, etc. Thus, this method presents a less strict definition of transgender. In future studies, an additional item asking participants to directly self-report their gender identity would facilitate a more precise classification.

Zucker^5^ has noted that it is important to consider the difficulties in determining the true prevalence of gender dysphoria in light of the influence of social factors. Masculine behaviors in individuals AFAB are more tolerated than feminine behavior in individuals AMAB. It is possible that transgender girls are less likely to disclose a transgender identity due to the social expectations of a masculine role. This hypothesis is consistent with this study’s results, which found a higher percentage of TGNC youth who were AFAB than TGNC who were AMAB.

Consistent with previous studies,^1,11,14^ the results of this study suggest that TGNC youth have relatively poor mental health status. Compared with the cisgender group, TGNC adolescents tended to have more health problems, including depression and anxiety symptoms. Among the gender identity minority groups, transgender girls had the highest risk of suicide attempts and were most likely to be bullied, nonbinary youth AMAB had the highest risk of having a suicide plan, transgender boys had the highest risk of having a self-harm plan, transgender boys and nonbinary youth AFAB had the highest risk of performing deliberate self-harm, and questioning youth AFAB had the highest risk of suicidal ideation. Similar to previous studies,^32,33,34^ compared with cisgender groups, TGNC adolescents were significantly more likely to have suicidal thoughts, suicide plans, and suicide attempts. Among the TGNC groups, transgender girls had the highest risk of a suicide attempt. This outcome was different from a US study by Toomey et al,^34^ which found that transgender boys had a higher rate of attempted suicide (50.8%) than transgender girls (29.9%). This finding could to due to the emphasis on masculine gender role expectations in Chinese culture and the deeply embedded stigma toward feminine males. This pattern seems to suggest that TGNC adolescents AFAB are more inclined to self-harm, whereas TGNC adolescents AMAB are more likely to attempt suicide.

School climate is important for the mental health of gender minority students, who experience discrimination in school settings (eg, gendered clothing requirements for school activities).^30,35^ One meta-analysis of longitudinal research showed that school victimization was closely associated with students’ psychological distress.^36^ Gender minority students often reported victimization and less school safety.^35^ Thus, social and educational implementation of gender identity support is important for reducing mental health problems.^1^

Our study supported claims that being bullied at school is a significant risk factor for self-harm behaviors and suicide attempts.^37^ In addition, our study also found that transgender girls, questioning AMAB, and nonbinary youth AMAB were more likely to report being bullied at school in comparison with cisgender adolescents. Conversely, within the TGNC group, transgender boys, nonbinary youth AFAB, and questioning youth AFAB had a much lower risk of being bullied at school. As previously mentioned, this outcome may reflect greater societal tolerance toward women behaving “manly” than men behaving “girly.”^5^ For the TGNC group, the vulnerability of bullying resulted from the sex-atypical expression of gender identity.^29^ This pattern may also reveal a cultural element in Chinese schools in which femininity is not encouraged. This attitude can be traced back to the Cultural Revolution era in China, illustrated in the famous Mao slogan, “The time has changed; men and women are the same.”^38^^p62^ In other words, both girls and boys in China, to some extent, are not encouraged to behave in a feminine manner. Indeed, there is a Chinese expression, *niang qiang,* that describes highly effeminate males, and the term almost always has negative connotations. It is important to further investigate the gender or sexual forms of bullying in order to identify its specific triggers.^29^

### Limitations

There were several limitations in the current study. First, due to the nature of the cross-sectional survey design, causality of the assessed factors cannot be confirmed. Second, it was difficult to determine the exact proportion of TGNC adolescents due to considerable variations in definitions and the method of sample estimation.^16^ It is possible that we had fewer transgender females than transgender males in the current sample because transgender females may identify and disclose their transgender identity at later ages. Participants’ interpretation of the gender identity questions could be affected by social and cultural factors. There is a possibility that participants could have interpreted the questions ambiguously. However, these questions have been used in European^21^ and Chinese populations^20^ before and have been tested as culturally appropriate for Chinese TGNC populations. Third, the data were collected in an economically developed region of China, which may not be generalizable to all Chinese TGNC adolescents. Fourth, this study investigated sensitive topics on gender minority status and suicidality. We used a school-based survey, and it is possible that adolescents have underreported on these topics. Moreover, the data were collected in schools, and adolescents excluded from schools were not accessed. Compared with the school sample, TGNC adolescents excluded from schools may have even more severe mental health issues. We suggest that future studies use large national samples in order to more rigorously estimate the size of the Chinese TGNC adolescent population.

## Conclusions

The current study results suggest poorer mental and physical conditions among TGNC adolescents compared with cisgender adolescents in China. The findings indicate the need for researchers, practitioners, and policy makers to address these mental health risks. School-level intervention is recommended to support the well-being and equity of gender minority youth.

## Notes

Supplement. **eTable.** Mixed-Effect Logistic Regression Results With Cisgender Girls Being the Reference Group: Associations Between Self-Harm, Suicidal Thoughts and Actions, and Being Bullied at School and Gender Groups
