# Supplementary material for: Mental Health Status of Cisgender and Gender-Diverse Secondary School Students in China
Source: JAMA Netw Open. 2020 Oct 27;3(10):e2022796. doi: 10.1001/jamanetworkopen.2020.22796 (PMC7592029; doi:10.1001/jamanetworkopen.2020.22796)
Supplement: Supplement. — eTable. Mixed-Effect Logistic Regression Results With Cisgender Girls Being the Reference Group: Associations Between Self-Harm, Suicidal Thoughts and Actions, and Being Bullied at School and Gender Groups [file jamanetwopen-e2022796-s001.pdf]

Supplementary Online Content

Wang Y, Yu H, Yang Y, et al. Mental health status of cisgender and gender-diverse secondary school students in China. *JAMA Netw Open*. 2020;3(10):e2022796. doi:10.1001/jamanetworkopen.2020.22796

eTable. Mixed Effect Logistic Regression Results With Cisgender Girls Being the Reference Group: Associations Between Self-Harm, Suicidal Thoughts and Actions, and Being Bullied at School and Gender Groups

This supplementary material has been provided by the authors to give readers additional information about their work.

© 2020 Wang Y et al. *JAMA Network Open.*

eTable 1. Mixed Effect Logistic Regression Results With Cisgender Girls Being the Reference Group: Associations Between Self-Harm, Suicidal Thoughts and Actions, and Being Bullied at School and Gender Groups

		n (No)	n (Yes)	YES %	Odds Ratio	95% CI of Odds Ratio	*p*
**Did you want to harm yourself in the last month?**							
	Cisgender boys	5151	634	10.96	0.68	[0.60, 0.76]	< .001
	Cisgender girls	3478	634	15.42	1.00		
	Transgender girls (AMAB)	149	56	27.31	2.08	[1.52, 2.84]	< .001
	Transgender boys (AFAB)	564	284	33.49	2.74	[2.35, 3.21]	< .001
	Nonbinary youth AMAB	100	35	25.92	1.93	[1.31, 2.86]	< .001
	Nonbinary youth AFAB	77	35	31.25	2.51	[1.66, 3.79]	< .001
	Questioning AMAB	236	77	24.60	1.77	[1.34, 2.33]	< .001
	Questioning AFAB	334	135	28.78	2.27	[1.83, 2.82]	< .001
**Did you deliberately harm yourself in the last month?**							
	Cisgender boys	5243	515	8.94	0.67	[0.60, 0.75]	< .001
	Cisgender girls	3578	526	12.82	1.00		
	Transgender girls (AMAB)	162	43	20.98	1.84	[1.29, 2.62]	< .001
	Transgender boys (AFAB)	652	196	23.11	2.05	[1.72, 2.45]	< .001
	Nonbinary youth AMAB	108	27	20.00	1.72	[1.11, 2.64]	.014
	Nonbinary youth AFAB	86	26	23.21	2.05	[1.31, 3.22]	.002
	Questioning AMAB	259	54	17.25	1.43	[1.05, 1.96]	.023
	Questioning AFAB	376	93	19.83	1.70	[1.34, 2.15]	< .001
**Did you think about suicide in the last month?**							

© 2020 Wang Y et al. *JAMA Network Open.*

	Cisgender boys	5158	623	10.78	0.70	[0.63, 0.79]	
	Cisgender girls	3501	598	14.59	1.00		< .001
	Transgender girls (AMAB)	139	66	32.20	2.77	[2.03, 3.79]	< .001
	Transgender boys (AFAB)	581	262	31.08	2.61	[2.19, 3.12]	< .001
	Nonbinary youth AMAB	97	37	27.61	2.22	[1.50, 3.29]	< .001
	Nonbinary youth AFAB	76	35	31.53	2.66	[1.76, 4.02]	< .001
	Questioning AMAB	238	74	23.72	1.79	[1.36, 2.35]	< .001
	Questioning AFAB	317	148	31.83	2.80	[2.26, 3.48]	< .001
**Did you have a suicide plan and prepare to die in the last month?**							
	Cisgender boys	5572	187	3.24	1.02	[0.81, 1.29]	.851
	Cisgender girls	3974	131	3.19	1.00		
	Transgender girls (AMAB)	179	26	12.68	4.53	[2.88, 7.10]	< .001
	Transgender boys (AFAB)	779	70	8.24	2.72	[2.03, 3.65]	< .001
	Nonbinary youth AMAB	115	20	14.81	5.47	[3.29, 9.11]	< .001
	Nonbinary youth AFAB	99	13	11.61	4.14	[2.25, 7.60]	< .001
	Questioning AMAB	296	17	5.43	1.77	[1.06, 2.94]	.031
	Questioning AFAB	434	34	7.26	2.44	[1.64, 3.60]	< .001
**Have you ever attempted suicide in the past?**							
	Cisgender boys	5538	217	3.77	0.86	[0.71, 1.05]	.162
	Cisgender girls	3928	177	4.31	1.00		
	Transgender girls (AMAB)	175	30	14.63	3.74	[2.48, 5.65]	< .001
	Transgender boys (AFAB)	761	87	10.26	2.51	[1.91, 3.30]	< .001
	Nonbinary youth AMAB	116	18	13.43	3.39	[2.03, 5.64]	< .001

© 2020 Wang Y et al. *JAMA Network Open.*

	Nonbinary youth AFAB	100	12	10.71	2.64	[1.44, 4.84]	.002
	Questioning AMAB	284	29	9.26	2.25	[1.49, 3.39]	< .001
	Questioning AFAB	436	33	7.04	1.66	[1.25, 2.46]	.009
**Have you ever been bullied at school in this academic year?**							
	Cisgender boys	5316	522	8.94	2.05	[1.72, 2.45]	
	Cisgender girls	3949	187	4.52	1.00		< .001
	Transgender girls (AMAB)	169	39	18.75	4.81	[3.31, 6.98]	< .001
	Transgender boys (AFAB)	779	81	9.42	2.16	[1.64, 2.84]	< .002
	Nonbinary youth AMAB	115	22	16.06	4.06	[2.53, 6.49]	< .003
	Nonbinary youth AFAB	104	8	7.14	1.62	[0.77, 3.40]	.202
	Questioning AMAB	265	51	16.14	4.01	[2.88, 5.60]	< .001
	Questioning AFAB	432	43	9.05	2.10	[1.47, 2.98]	< .001

Note. School was added as a random effect term. $p < .01$ as the cutoff criterion.

© 2020 Wang Y et al. *JAMA Network Open.*
